# 6-Chloro-4-(2-phenyl­ethen­yl)chroman-2-one

**DOI:** 10.1107/S1600536810045101

**Published:** 2010-11-06

**Authors:** Kwang-Su Choi, Sung-Gon Kim

**Affiliations:** a Department of Chemistry, Kyonggi University, San 94-6, Iui-dong, Yeongtong-gu, Suwon 443-760, Republic of Korea

## Abstract

The title compound, C_17_H_13_ClO_2_, was obtained from the oxidation of 6-chloro-4-(2-phenyl­ethen­yl)chroman-2-ol, which was synthesized by the reaction of of (*E*)-3-(5-chloro-2-hy­droxy­phen­yl)acryl­aldehyde with styrylboronic acid using diethyl­amine as a catalyst. The six-membered pyran­one ring of the chromane system has a screw-boat conformation. The dihedral angle between the least-squares planes of the chromane ring system and the styryl group is 85.28 (9)°.

## Related literature

For the synthesis of the title compound, see: Choi & Kim (2010 ▶). For the biological activity of chromenes, see: Ellis & Lockhart (2007 ▶); Green *et al.* (1996) ▶; Horton *et al.* (2003 ▶).
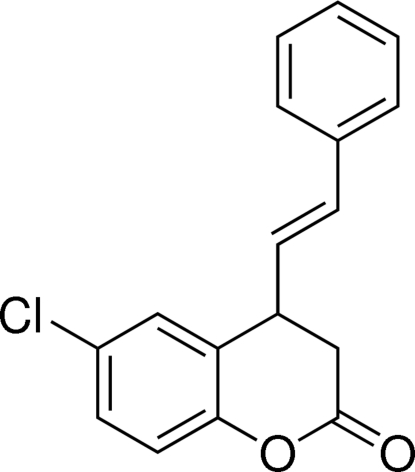


## Experimental

### 

#### Crystal data

C_17_H_13_ClO_2_
*M* *_r_* = 284.72
Monoclinic, 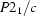

*a* = 15.6682 (3) Å
*b* = 6.2800 (1) Å
*c* = 14.9383 (3) Å
β = 115.129 (1)°
*V* = 1330.76 (4) Å^3^
*Z* = 4
Mo *K*α radiation
μ = 0.29 mm^−1^
*T* = 100 K
0.28 × 0.13 × 0.05 mm

#### Data collection

Bruker APEXII CCD diffractometer
Absorption correction: multi-scan (*SADABS*; Bruker, 2005 ▶) *T*_min_ = 0.925, *T*_max_ = 0.986
12258 measured reflections
3325 independent reflections
2839 reflections with *I* > 2σ(*I*)
*R*_int_ = 0.021

#### Refinement

*R*[*F* ^2^ > 2σ(*F* ^2^)] = 0.033
*wR*(*F* ^2^) = 0.083
*S* = 1.06
3325 reflections
181 parameters
H-atom parameters constrained
Δρ_max_ = 0.37 e Å^−3^
Δρ_min_ = −0.25 e Å^−3^

### 

Data collection: *APEX2* (Bruker, 2005 ▶); cell refinement: *SAINT* (Bruker, 2005 ▶); data reduction: *SAINT*; program(s) used to solve structure: *SHELXS97* (Sheldrick, 2008 ▶); program(s) used to refine structure: *SHELXL97* (Sheldrick, 2008 ▶); molecular graphics: *SHELXTL* (Sheldrick, 2008 ▶); software used to prepare material for publication: *SHELXTL*.

## Supplementary Material

Crystal structure: contains datablocks I, global. DOI: 10.1107/S1600536810045101/is2627sup1.cif

Structure factors: contains datablocks I. DOI: 10.1107/S1600536810045101/is2627Isup2.hkl

Additional supplementary materials:  crystallographic information; 3D view; checkCIF report

## Figures and Tables

**Fig. 1.**
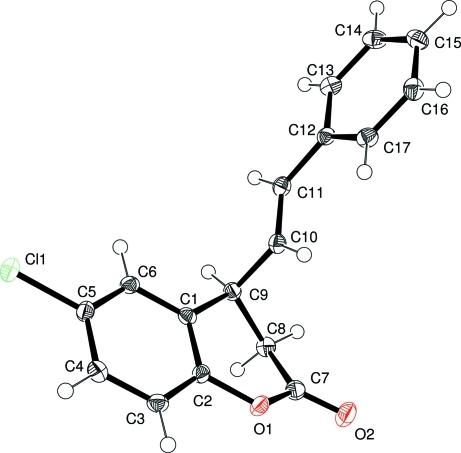
A view of the molecular structure of the title compound, showing the atom-numbering scheme. Displacement ellipsoids are drawn at the 50% probability level.

**Fig. 2.**
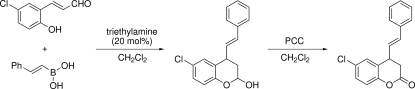
The preparation scheme of the title compound.

## supplementary crystallographic information

### Comment

Chromanes (dihydrobenzopyranes) are ubiquitously found in numerous biologically active natural products. Molecules containing chromane scaffolds exhibit a broad range of bioactivities, such as antiviral, antitumor, antimicrobial, sex pheromone, and those of the central nervous system activity (Ellis & Lockhart, 2007; Green *et al.*, 1996; Horton *et al.* 2003). We report herein the crystal structure of the title compound, which belongs to this class of compounds.

In the title compound, the six-membered pyranone ring of the chromane system has a screw-boat conformation. The dihedral angle between the least-squares planes of the chromane ring system and the styryl group is 85.28 (9)°.

### Experimental

To a solution of triethylamine (0.10 mmol) in CH_2_Cl_2_ (1.5 ml) was added styrylboronic acid (0.60 mmol) at room temperature. The solution was stirred for 5 min before addition of (*E*)-3-(5-chloro-2-hydroxyphenyl)acrylaldehyde (0.50 mmol). After stirring for 3 h, the resulting mixture was direct purified by silica gel chromatography to afford 6-chloro-3,4-dihydro-4-styryl-2*H*-chromen-2-ol. Oxidation of 6-chloro-3,4-dihydro-4-styryl-2*H*-chromen-2-ol (0.40 mmol) was performed in CH_2_Cl_2_ (2.0 ml) by adding of pyridinium chlorochromate (0.40 mmol) at room temperature. After 3 h, additional pyridinium chlorochromate (0.40 mmol) was added and after 6 h purification by silica gel chromatography was afforded the title compound (Fig. 2). Crystals suitable for X-ray analysis were obtained by slow evaporation from an *n*-hexane/CH_2_Cl_2_ solution.

### Refinement

All H atoms were positioned geometrically, with C—H = 0.93–0.98 Å and constrained to ride on their parent atoms, with *U*_iso_(H) = *xU*_eq_(C), where *x* = 1.5 for methyl H and *x* = 1.2 for all other H atoms.

### Crystal data

**Table:**

C_17_H_13_ClO_2_	*F*(000) = 592
*M**_r_* = 284.72	*D*_x_ = 1.421 Mg m^−^^3^
Monoclinic, *P*2_1_/*c*	Mo *K*α radiation, λ = 0.71073 Å
Hall symbol: -P 2ybc	Cell parameters from 5561 reflections
*a* = 15.6682 (3) Å	θ = 3.6–28.3°
*b* = 6.2800 (1) Å	µ = 0.28 mm^−^^1^
*c* = 14.9383 (3) Å	*T* = 100 K
β = 115.129 (1)°	Block, silver
*V* = 1330.76 (4) Å^3^	0.28 × 0.13 × 0.05 mm
*Z* = 4	

### Data collection

**Table:**

Bruker APEXII CCD diffractometer	3325 independent reflections
Radiation source: fine-focus sealed tube	2839 reflections with *I* > 2σ(*I*)
graphite	*R*_int_ = 0.021
φ and ω scans	θ_max_ = 28.4°, θ_min_ = 1.4°
Absorption correction: multi-scan (*SADABS*; Bruker, 2005)	*h* = −18→20
*T*_min_ = 0.925, *T*_max_ = 0.986	*k* = −8→8
12258 measured reflections	*l* = −19→14

### Refinement

**Table:**

Refinement on *F*^2^	Primary atom site location: structure-invariant direct methods
Least-squares matrix: full	Secondary atom site location: difference Fourier map
*R*[*F*^2^ > 2σ(*F*^2^)] = 0.033	Hydrogen site location: inferred from neighbouring sites
*wR*(*F*^2^) = 0.083	H-atom parameters constrained
*S* = 1.06	*w* = 1/[σ^2^(*F*_o_^2^) + (0.0306*P*)^2^ + 0.755*P*] where *P* = (*F*_o_^2^ + 2*F*_c_^2^)/3
3325 reflections	(Δ/σ)_max_ = 0.001
181 parameters	Δρ_max_ = 0.37 e Å^−^^3^
0 restraints	Δρ_min_ = −0.24 e Å^−^^3^

### Special details

**Table:**

Geometry. All e.s.d.'s (except the e.s.d. in the dihedral angle between two l.s. planes) are estimated using the full covariance matrix. The cell e.s.d.'s are taken into account individually in the estimation of e.s.d.'s in distances, angles and torsion angles; correlations between e.s.d.'s in cell parameters are only used when they are defined by crystal symmetry. An approximate (isotropic) treatment of cell e.s.d.'s is used for estimating e.s.d.'s involving l.s. planes.
Refinement. Refinement of *F*^2^ against ALL reflections. The weighted *R*-factor *wR* and goodness of fit *S* are based on *F*^2^, conventional *R*-factors *R* are based on *F*, with *F* set to zero for negative *F*^2^. The threshold expression of *F*^2^ > σ(*F*^2^) is used only for calculating *R*-factors(gt) *etc*. and is not relevant to the choice of reflections for refinement. *R*-factors based on *F*^2^ are statistically about twice as large as those based on *F*, and *R*- factors based on ALL data will be even larger.

### Fractional atomic coordinates and isotropic or equivalent isotropic displacement parameters (Å^2^)

**Table:**

	*x*	*y*	*z*	*U*_iso_*/*U*_eq_	
Cl1	0.64583 (2)	0.83763 (5)	0.61691 (2)	0.02134 (9)	
O1	0.35931 (6)	0.24736 (14)	0.62698 (7)	0.0185 (2)	
O2	0.21611 (7)	0.17409 (16)	0.60995 (8)	0.0267 (2)	
C1	0.39949 (9)	0.6045 (2)	0.59464 (9)	0.0149 (2)	
C2	0.42529 (9)	0.3975 (2)	0.62766 (9)	0.0156 (2)	
C3	0.51713 (9)	0.3250 (2)	0.65950 (9)	0.0176 (3)	
H3A	0.5325	0.1864	0.6827	0.021*	
C4	0.58581 (9)	0.4603 (2)	0.65657 (9)	0.0186 (3)	
H4A	0.6477	0.4141	0.6777	0.022*	
C5	0.56024 (9)	0.6657 (2)	0.62152 (9)	0.0166 (3)	
C6	0.46877 (9)	0.7392 (2)	0.59102 (9)	0.0161 (2)	
H6A	0.4536	0.8781	0.5682	0.019*	
C7	0.27232 (9)	0.3112 (2)	0.61886 (10)	0.0186 (3)	
C8	0.25822 (9)	0.5461 (2)	0.62445 (10)	0.0181 (3)	
H8A	0.2881	0.5907	0.6931	0.022*	
H8B	0.1913	0.5753	0.5998	0.022*	
C9	0.29900 (9)	0.6771 (2)	0.56459 (9)	0.0158 (2)	
H9A	0.2998	0.8275	0.5824	0.019*	
C10	0.24087 (9)	0.6545 (2)	0.45496 (9)	0.0163 (2)	
H10A	0.2362	0.5208	0.4265	0.020*	
C11	0.19575 (9)	0.8170 (2)	0.39674 (10)	0.0170 (3)	
H11A	0.2035	0.9496	0.4269	0.020*	
C12	0.13500 (9)	0.8094 (2)	0.28984 (9)	0.0158 (2)	
C13	0.07977 (9)	0.9877 (2)	0.24476 (10)	0.0189 (3)	
H13A	0.0856	1.1106	0.2816	0.023*	
C14	0.01637 (9)	0.9834 (2)	0.14573 (10)	0.0213 (3)	
H14A	−0.0200	1.1029	0.1169	0.026*	
C15	0.00700 (9)	0.8019 (2)	0.08960 (10)	0.0209 (3)	
H15A	−0.0367	0.7976	0.0238	0.025*	
C16	0.06374 (9)	0.6256 (2)	0.13271 (10)	0.0202 (3)	
H16A	0.0591	0.5048	0.0949	0.024*	
C17	0.12697 (9)	0.6293 (2)	0.23151 (10)	0.0184 (3)	
H17A	0.1645	0.5109	0.2594	0.022*	

### Atomic displacement parameters (Å^2^)

**Table:**

	*U*^11^	*U*^22^	*U*^33^	*U*^12^	*U*^13^	*U*^23^
Cl1	0.01884 (16)	0.02132 (17)	0.02491 (18)	−0.00529 (12)	0.01029 (13)	−0.00125 (13)
O1	0.0182 (4)	0.0133 (4)	0.0248 (5)	−0.0015 (4)	0.0099 (4)	0.0013 (4)
O2	0.0231 (5)	0.0219 (5)	0.0368 (6)	−0.0055 (4)	0.0142 (5)	0.0006 (4)
C1	0.0160 (6)	0.0155 (6)	0.0117 (6)	0.0004 (5)	0.0044 (5)	−0.0005 (5)
C2	0.0172 (6)	0.0153 (6)	0.0138 (6)	−0.0027 (5)	0.0062 (5)	−0.0011 (5)
C3	0.0196 (6)	0.0145 (6)	0.0169 (6)	0.0023 (5)	0.0061 (5)	0.0020 (5)
C4	0.0156 (6)	0.0203 (6)	0.0180 (6)	0.0011 (5)	0.0053 (5)	−0.0005 (5)
C5	0.0173 (6)	0.0174 (6)	0.0156 (6)	−0.0044 (5)	0.0073 (5)	−0.0019 (5)
C6	0.0198 (6)	0.0136 (6)	0.0138 (6)	−0.0009 (5)	0.0060 (5)	0.0000 (5)
C7	0.0188 (6)	0.0209 (7)	0.0168 (6)	−0.0015 (5)	0.0081 (5)	0.0003 (5)
C8	0.0177 (6)	0.0191 (6)	0.0187 (6)	0.0002 (5)	0.0087 (5)	−0.0008 (5)
C9	0.0160 (6)	0.0134 (6)	0.0169 (6)	0.0003 (5)	0.0059 (5)	−0.0006 (5)
C10	0.0154 (6)	0.0153 (6)	0.0176 (6)	−0.0010 (5)	0.0064 (5)	−0.0020 (5)
C11	0.0160 (6)	0.0166 (6)	0.0195 (6)	−0.0009 (5)	0.0087 (5)	−0.0008 (5)
C12	0.0137 (5)	0.0173 (6)	0.0173 (6)	−0.0005 (5)	0.0076 (5)	0.0027 (5)
C13	0.0211 (6)	0.0175 (6)	0.0214 (7)	0.0022 (5)	0.0123 (5)	0.0013 (5)
C14	0.0206 (6)	0.0235 (7)	0.0218 (7)	0.0074 (5)	0.0110 (5)	0.0069 (5)
C15	0.0170 (6)	0.0297 (7)	0.0158 (6)	0.0015 (5)	0.0067 (5)	0.0029 (5)
C16	0.0200 (6)	0.0216 (7)	0.0201 (7)	−0.0018 (5)	0.0096 (5)	−0.0031 (5)
C17	0.0176 (6)	0.0167 (6)	0.0217 (7)	0.0031 (5)	0.0092 (5)	0.0030 (5)

### Geometric parameters (Å, °)

**Table:**

Cl1—C5	1.7456 (13)	C9—C10	1.5049 (17)
O1—C7	1.3758 (15)	C9—H9A	0.9800
O1—C2	1.3962 (15)	C10—C11	1.3322 (18)
O2—C7	1.1981 (16)	C10—H10A	0.9300
C1—C2	1.3880 (18)	C11—C12	1.4722 (18)
C1—C6	1.3953 (17)	C11—H11A	0.9300
C1—C9	1.5127 (17)	C12—C13	1.4002 (18)
C2—C3	1.3867 (18)	C12—C17	1.4004 (18)
C3—C4	1.3863 (18)	C13—C14	1.3884 (19)
C3—H3A	0.9300	C13—H13A	0.9300
C4—C5	1.3861 (19)	C14—C15	1.385 (2)
C4—H4A	0.9300	C14—H14A	0.9300
C5—C6	1.3856 (18)	C15—C16	1.3945 (19)
C6—H6A	0.9300	C15—H15A	0.9300
C7—C8	1.4990 (19)	C16—C17	1.3851 (19)
C8—C9	1.5394 (17)	C16—H16A	0.9300
C8—H8A	0.9700	C17—H17A	0.9300
C8—H8B	0.9700		
			
C7—O1—C2	120.43 (10)	C10—C9—C8	111.91 (10)
C2—C1—C6	117.87 (11)	C1—C9—C8	107.64 (10)
C2—C1—C9	119.80 (11)	C10—C9—H9A	108.6
C6—C1—C9	122.32 (11)	C1—C9—H9A	108.6
C3—C2—C1	122.19 (12)	C8—C9—H9A	108.6
C3—C2—O1	115.96 (11)	C11—C10—C9	123.12 (12)
C1—C2—O1	121.80 (11)	C11—C10—H10A	118.4
C4—C3—C2	119.62 (12)	C9—C10—H10A	118.4
C4—C3—H3A	120.2	C10—C11—C12	127.08 (12)
C2—C3—H3A	120.2	C10—C11—H11A	116.5
C5—C4—C3	118.59 (12)	C12—C11—H11A	116.5
C5—C4—H4A	120.7	C13—C12—C17	118.20 (12)
C3—C4—H4A	120.7	C13—C12—C11	118.61 (12)
C6—C5—C4	121.80 (12)	C17—C12—C11	123.15 (12)
C6—C5—Cl1	119.05 (10)	C14—C13—C12	120.90 (12)
C4—C5—Cl1	119.14 (10)	C14—C13—H13A	119.6
C5—C6—C1	119.89 (12)	C12—C13—H13A	119.6
C5—C6—H6A	120.1	C15—C14—C13	120.31 (13)
C1—C6—H6A	120.1	C15—C14—H14A	119.8
O2—C7—O1	117.02 (12)	C13—C14—H14A	119.8
O2—C7—C8	126.49 (12)	C14—C15—C16	119.39 (13)
O1—C7—C8	116.47 (11)	C14—C15—H15A	120.3
C7—C8—C9	112.71 (10)	C16—C15—H15A	120.3
C7—C8—H8A	109.0	C17—C16—C15	120.39 (12)
C9—C8—H8A	109.0	C17—C16—H16A	119.8
C7—C8—H8B	109.0	C15—C16—H16A	119.8
C9—C8—H8B	109.0	C16—C17—C12	120.75 (12)
H8A—C8—H8B	107.8	C16—C17—H17A	119.6
C10—C9—C1	111.54 (10)	C12—C17—H17A	119.6
			
C6—C1—C2—C3	2.07 (19)	C2—C1—C9—C10	−94.72 (14)
C9—C1—C2—C3	−177.61 (12)	C6—C1—C9—C10	85.61 (14)
C6—C1—C2—O1	−175.28 (11)	C2—C1—C9—C8	28.41 (15)
C9—C1—C2—O1	5.04 (18)	C6—C1—C9—C8	−151.26 (12)
C7—O1—C2—C3	165.08 (11)	C7—C8—C9—C10	72.17 (14)
C7—O1—C2—C1	−17.41 (17)	C7—C8—C9—C1	−50.73 (14)
C1—C2—C3—C4	−1.5 (2)	C1—C9—C10—C11	−122.70 (13)
O1—C2—C3—C4	175.98 (11)	C8—C9—C10—C11	116.65 (13)
C2—C3—C4—C5	−0.02 (19)	C9—C10—C11—C12	−177.86 (11)
C3—C4—C5—C6	0.96 (19)	C10—C11—C12—C13	168.28 (12)
C3—C4—C5—Cl1	−179.75 (10)	C10—C11—C12—C17	−9.2 (2)
C4—C5—C6—C1	−0.38 (19)	C17—C12—C13—C14	2.21 (18)
Cl1—C5—C6—C1	−179.67 (9)	C11—C12—C13—C14	−175.41 (11)
C2—C1—C6—C5	−1.11 (18)	C12—C13—C14—C15	−0.25 (19)
C9—C1—C6—C5	178.56 (11)	C13—C14—C15—C16	−1.82 (19)
C2—O1—C7—O2	173.59 (12)	C14—C15—C16—C17	1.90 (19)
C2—O1—C7—C8	−7.79 (17)	C15—C16—C17—C12	0.10 (19)
O2—C7—C8—C9	−138.65 (14)	C13—C12—C17—C16	−2.13 (18)
O1—C7—C8—C9	42.87 (16)	C11—C12—C17—C16	175.38 (12)
